# Neopterin: Biomarker of cell-mediated immunity and potent usage as biomarker in silicosis and other occupational diseases

**DOI:** 10.4103/0019-5278.44690

**Published:** 2008-12

**Authors:** Shubhangi K. Pingle, Rajani G. Tumane, Aruna A. Jawade

**Affiliations:** National Institute of Miner's Health, JNARDDC Campus, Nagpur, India

**Keywords:** Biomarker, cell-mediated immunity, γ-interferon, neopterin, silicosis, tryptophan degradation

## Abstract

Neopterin is regarded as an early biomarker of the cellular immune response. This low molecular mass compound belongs to the class of pteridine and is a metabolite of guanosine triphosphate, which is produced by the activated macrophages and dendritic cells after stimulation with γ-interferon. An international group acknowledges the fact that the levels of serum neopterin can be used as a marker of the effect of exposure to silica and other occupational diseases. The determination of neopterin is an innovative tool for monitoring diseases associated with the activation of cell-mediated immunity.

## INTRODUCTION

Neopterin [D-erythro-6-(1′,2′,3′-trihydroxypropyl)-pterin][1] is known to be in equilibrium with 7,8-dihydroneopterin, and the presence of a high concentration of both oxidized and reduced forms of pteridin may be associated with oxidative stress. Silica is one of the most documented contaminants of the work place. Long-term occupational exposure to silica is associated with an increased risk for respiratory diseases such as silicosis, tuberculosis, chronic bronchitis, chronic obstructive pulmonary disease and lung cancer.[2] Furthermore, a variety of immune dysfunction-related diseases have been reported in the silicotic individual. Preliminary studies indicating an enhanced level of autoantibody and several cytokines reflect an involvement of the immune system in the pathogenesis of silicosis and the resulting complication as an early and valuable marker of cellular immunity.[2] Neopterin levels can be used as a marker of the effect of exposure to silica.[3]

Neopterin is pyrazino–pyrimidine compound of molecular weight 253 D belonging to the class of pteridines. Pteridine is a chemical compound of fused pyrimidine and pyrizine rings. A pteridine is a group of heterocyclic compounds containing a wide variety of substitutions in this structure.[4] Pterins and flavins are a class of substituted pteridines that have important biological activities.[4] It is produced by guanosine triphosphate (GTP) via γ-interferon (INF-γ) following the activation of T cells. Neopterin concentration increase in the urine or blood reflects the activation of cellular immunity and an endogenous release of INF-γ.[5] This review focuses on the clinical utility of measuring the neopterin levels in inflammatory diseases and the potential functions of neopterin as a mediator and modulator in the course of inflammatory and infectious processes. *In vitro* studies reveal that neopterin derivatives exhibit distinct biochemical effects, most likely via interactions with reactive oxygen or nitrogen intermediates, thereby affecting the cellular redox state.[5] Neopterin enhances the cytotoxic potential of the activated macrophages (AC) and the dendritic cells (DC). In vivo, a strong correlation was obtained between the neopterin levels and the disease severity, progression and outcome of infections with inflammatory disease. The influence of neopterin derivatives on the cellular metabolism may provide an explanation for these clinical observations.

## MECHANISM OF NEOPTERIN ACTIVATION

**Figure 1:**
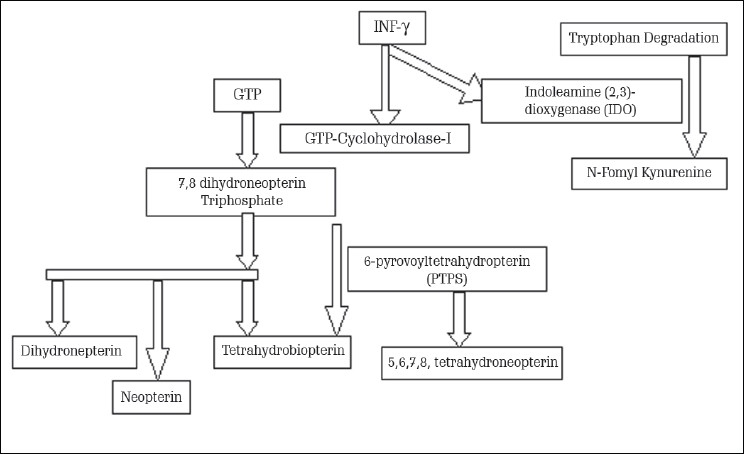
Mechanism of Neopterin Activation

Neopterin is derived from GTP and is produced by stimulated macrophages under the influence of INF-γ of lymphocyte origin. Cleavage of GTP by GTP-cyclohydrolase I results in neopterin, yielding 7,8-dihydroneopterin triphosphate, which is a joint precursor of dihydroneopterin, neopterin, tetrahydrobiopterin, a necessary cofactor of aromatic amino acid monooxygenase, and nitric oxide synthases (NOS). Human monocytes/macrophages are the unique source to produce an excess of neopterin derivatives at the rate of 5, 6, 7, 8-tetrahydrobiopterin,[6] which results as a comparably low activity of 6-pyrovoyltetrahydropterin synthase, which is the first enzyme in the conversion of 7,8-dihydroneopterin triphosphate to tetrahydrobiopterin.[7] On activation of the cellular immunity, IFN-γ induces GTP-cyclohydrolase I and also stimulates the enzyme indoleamine (2, 3)-dioxygenase (IDO) in various cells.[8,9] In tryptophan catabolism, N-formyl-kynurenine, the first intermediate, is formed in response to IDO within the biosynthetic pathway of nicotinamide dinucleotide. To monitor the activation status of IDO and of cellular immunity, determination of kynurenine and tryptophan concentrations has proven to be a sensitive estimate both in vivo and in vitro[10,11] [Figure 1].

## OXIDATIVE STRESS AND NEOPTERIN

Recent data suggest that neopterin derivatives exhibit distinct biochemical functions. Neopterin was found to enhance the effects of toxic reactive oxygen species originating from chloramine T and hydrogen peroxide,[12,13] suggesting that neopterin derivatives are able to modulate macrophage-induced cytotoxicity by the induction of oxidative stress. In rat vascular smooth muscle cells, neopterin stimulates redox-sensitive intracellular signal transduction cascades, thereby triggering the inducible NOS (iNOS) gene expression at the messenger ribonucleic acid level with a subsequent increase in nitric oxide (NO) production.[14] In vascular muscle cells[15] and Jurkat cells,[16] the neopterin derivatives were found to activate the transcriptional nuclear factor (NF)-κB. It has been shown to induce programmed cell death, which is mediated by the reactive oxygen intermediates in T-lymphoblastic cell lines and in rat alveolar cells.[15,16] In regard to these multiple biochemical functions of neopterin derivatives, it is very likely that DC might also use neopterin derivatives in the regulation of T cell response. Uniquely, in humans and primates, high concentrations of neopterin are detected during cellular immune activation produced by AM and also by DC. In all other organisms, activation of cellular immunity is accompanied by an increased production of tetrahydrobiopterin, an essential cofactor for iNOS. NO production by iNOS seems to play a role in inflammation, e.g., by acting regulatorily on NF-_κ_B, an important modulator of inflammatory gene expression, including pro-inflammatory cytokines and endothelial cell adhesion molecules.[17] The production of neopterin derivatives instead of biopterin derivatives in humans suggest that neopterin derivatives substitute regulatory and immunological functions, especially of the tetrahydrobiopterin-induced NO generation.

DC produces neopterin derivatives on stimulation, which is additionally determined by the degree of IDO-dependent tryptophan degradation. As observed, neopterin production and IDO activation were found to closely correlate in a large variety of diseases in vivo, including systemic lupus erythematosus, hepatitis immunodeficiency virus and in pregnancy.[10,18,19]

## ACTIVATION OF NEOPTERIN BY INF AFFECTS THE IMMUNE SYSTEM

To activate the gene transcription by initiation of intracellular signaling via a complex mechanism, INF binds to specific cell surface receptors. The gene gets modulated by INF stimulation and effects on inhibition of viral replication, cell proliferation and immunomodulation in infected cells. An effector protein such as neopterin and 2′, 5′-oligoadeylates synthetase gets stimulated by INF. Neopterin has been used in the INF studies to demonstrate its immune activation by the INF. Neopterin has been validated in a large number of studies as a marker of INF activation. The level of neopterin increases with the INF.[20]

## NEUROTRANSMITTERS AND NEOPTERIN

Occupational exposure to lead affects the neuromuscular junctions and it might cause disturbances in the locomotor activity. This study was carried out to evaluate pteridine metabolism for the synthesis of neurotransmitters in urinary neopterin, biopterin, creatinine and blood dihydropteridine reductase (DHPR) activity in battery workers and the delta-aminolevulonic acid (delta-ALA) was measured. Blood and urine lead levels were detected by an atomic absorption spectrophotometer. A significant increase in the blood and urine lead levels shows traces of urinary neopterin, biopterin and delta-ALA among exposed workers.[21] DHPR activity was indifferent as compared with the control group. These studies demonstrate that an increased activity of the pteridine pathway causes accumulation of the neurotransmitters, which may be responsible for the neurological disorders.[22]

## NEOPTERIN AND DIFFERENT DISEASES

It has been suggested that it is an excellent marker for the activation of the monocyte/macrophage axis in some clinical situations. Increased amounts of neopterin in body fluids are associated with a variety of diseases in which activation of the cellular immune mechanism is involved, such as certain malignancies, allograft rejection, autoimmune diseases and viral infections.[1,23–25] Elevated neopterin levels were observed in silicotic individuals,[2,26] rheumatoid arthritis (RA),[27] neuropsychiatric abnormalities,[28] Kaposi's sarcoma,[29] intrahepatic cholestasis of pregnancy,[30] pulmonary tuberculosis and follow-up of antituberculosis treatment,[31] activation of cell-mediated immunity (CMI) during pregnancy[32] and severe burn sepsis.[33] Neopterin and its reduced form modulate the cytotoxic substances, and it also leads to the generation of singlet oxygen, hydroxyl radical and NO.[12,34] [Table 1]

**Table 1:** Neopterin and different diseases

Reference	Title	Number of subjects & Study design	Results			Comments
Altindag *et. al*.[^2^]	Neopterin is a new biomarker for the evaluation of occupational exposure to silica	Serum and urinary neopterin level investigated in silica exposed (n=22) and healthy volunteers (n=20) investigated by ELISA spectrophotometry & HPLC technique	Elevated levels of Neopterin in serum and urine were observed.			Neopterin can be used for biological monitoring in workplaces for clinical diagnosis and prognosis.
				**Control**	**Exposed patient**	
			Serum	5.98±0.44 nmol/L	7.89±1.97nmol/L	
			Urine	97.60±41.42μmol/mol creatinine	165.59±78.20μmol/mol creatinine
Prakava, *et. al*.[^26^]	The potential role of neopterin as a biomarker for silicosis.	Serum Neopterin concentration measured in 60 patients with silicosis, according to conventional X-ray observation (ILO 2002). Investigation on 84 RA patients was performed	The serum Neopterin level significantly higher as compared to control group.			The increased serum neopterin concentration could be used as a biomarker for silicosis.
			**Control**	**Exposed patient**		
			1.56±0.39 ng/ml	2.74±1.12ng/ml		
Kullich *et. al*.	Correlation of interleukin-2 receptor and neopterin secretion in rheumatoid arthritis (RA).		The significant correlation between neopterin and IL-2R in RA points out a possible connection in mechanisms of the immune system			In rheumatoid arthritis (RA) T-lymphocytes, the secretion of interleukin-2 and its receptor (IL-2R) is influenced
Santelli *et. al*.[^29^]	Urinary neopterin and immunological feature in patient with kaposi's Sarcoma	Neopterin excretion level and immunological features of 20 patient of CKS and 30 normal control	Classical form of CKS increases neopterin level were as significant reduction in CD+3, CD+4 lymphocytes. CD+8 did not show significant variation. Increase IgA are observed.			These finding seems to confirm CKS as an opportunistic neoplasia indicate neopterin as a prognostic marker.
Wang. *et. al*.[^30^]	Increased serum levels of neopterin and soluble Interleukin-2 receptor (sIL-2R), in ICP.	30 patients with ICP and 30 healthy pregnant ladies serum levels assayed for neopterin and sIL-2R.	Serum levels of Neopterin and sIL-2R were increase significantly in women with ICP			Activation of monocytes macrophage and lymphocyte demonstrated in ICP.
Ghonaim *et. al*.[^31^]	Soluble Interleukin- 2 Receptor Alpha (sIL-2Rα) and Neopterin In Patient With Pulmonary Tuberculosis	The study included 44 patients with active pulmonary TB and 20 controls. Serum levels of sIL-2Rα and neopterin were determined.	Significantly elevated levels of sIL-2Rα observed in TB patient with active diseases (1253±492pgm/ml) as compared to controls (412±173pg/ml) and the neopterin level observed also higher in active TB (45.8±17.1nmol/L) than controls (5.2±1.8nmol/L).	sIL-2Rα and neopterin serum level are increased in patient with pulmonary TB and can be used as markers for disease activity and follow up of anti-tuberculosis treatment.
Fuith *et. al*.[^32^]	Neopterin, a marker of cell mediated immune activation in human pregnancy.	Serum and urine samples were studied for neopterin and INF-γ concentration in normal pregnant women.	Neopterin concentration exceeded the normal range in (79%) of pregnant ladies. Neopterin levels increase with the time of pregnancy. However no circulation of INF-γ was detected.	Neopterin levels provide evidences for activation of cell mediated immunity during pregnancy.
Yao *et. al*.[^33^]	Elevation serum neopterin level: its relation to endotoxaemia and sepsis in patient with major burns.	This prospective study included 35 patients with burn size greater than 30% and 22 healthy volunteers.	High neopterin level was found in patient with sepsis and the marked elevation persists. Patient with endotoxaemia had much high neopterin concentration.	High neopterin levels associated with a critical event in the development of severe burn sepsis.
Weiss *et. al*.[^12^]	Neopterin modulates toxicity mediated by reactive oxygen and chloride species.	This study performed to test the ability of neopterin and its reduced form to modulate the effect of cytotoxic substances like hydrogen peroxide or hypochlorous acid and N-chloramine derivatives.	Observations showed that 7,8-dihydroneopterin potentially reduces biological and chemical effects of these substances independently from the pH value. In contrast, at slightly alkaline pH (pH 7.5) neopterin enhances hydrogen peroxide and chloramine-T activity.	Neopterin is able both to enhance and to reduce the cytotoxicity in dependence of pH value and its oxidation state, and it may have a pivotal role in modulation of macrophage mediated effector mechanism.
Razumovitch *et. al*,[^34^]	Influence of neopterin on the generation of reactive oxygen species (ROS) in human neutrophils	The study performs the influence of neopterin on the generation of ROS in neutrophils suspended in Earl's solution by monitoring of biochemiluminometer.	Neopterin induced luminance in suspensions of neutrophils in the presence of luminal, but not in lucigenin and also observed that neopterin affect the adhesive cells.	Neutrophils respond on exposure to neopterin with additional generation of singlet oxygen, hydroxyl radical and nitric oxide.

## CONCLUSION

The most important clinical application of the determination of neopterin is as a prognostic indicator and as a follow-up for chronic infection, immune stimulation monitoring, differential diagnosis of acute bacterial and viral infection and also as an early indication for the complication of allograft recipient.[23,24] As the neopterin level serves as an indirect indicator for oxidative stress, the CMI marker, the levels of neopterin help in the diagnosis of different diseases.[5] Elevated levels in the serum of the silicotic patients and the correlation of baseline neopterin with IFN will help in more advances in occupational diseases.[35] In RA, an immune dysregulation alters the release of neopterin from human monocytes/macrophages.[27] The increased formation of neopterin and degradation of tryptophan may result in a decreased T cell response along with the development of “immunodeficiency”.[36] Pteridins are used as biochemical markers of immune system activation, mainly the markers of early activation and development of CMI response, which are used for the evaluation of pathologic disturbances. From the above studies, it may be concluded that elevated levels of neopterin can be used as a biomarker of CMI, silicosis and other occupational diseases.
